# Differences in DNA Methylation Between Disease-Resistant and Disease-Susceptible Chinese Tongue Sole (*Cynoglossus semilaevis*) Families

**DOI:** 10.3389/fgene.2019.00847

**Published:** 2019-09-13

**Authors:** Yunji Xiu, Changwei Shao, Ying Zhu, Yangzhen Li, Tian Gan, Wenteng Xu, Francesc Piferrer, Songlin Chen

**Affiliations:** ^1^Key Lab of Sustainable Development of Marine Fisheries, Ministry of Agriculture; Yellow Sea Fisheries Research Institute, Chinese Academy of Fishery Sciences, Qingdao, China; ^2^Laboratory for Marine Fisheries Science and Food Production Processes, Qingdao National Laboratory for Marine Science and Technology, Qingdao, China; ^3^School of Marine Science and Engineering, Qingdao Agricultural University, Qingdao, China; ^4^Institut de Ciències del Mar (ICM), Spanish National Research Council (CSIC), Barcelona, Spain

**Keywords:** DNA methylation, whole-genome bisulfite sequencing, *Cynoglossus semilaevis*, disease-resistant, disease-susceptible

## Abstract

DNA methylation, the most widely studied and most well-understood epigenetic modification, has been reported to play crucial roles in diverse processes. Although it has been found that DNA methylation can modulate the expression of immune-related genes in teleosts, a systemic analysis of epigenetic regulation on teleost immunity has rarely been performed. In this research, we employed whole-genome bisulfite sequencing to investigate the genome-wide DNA methylation profiles in select disease-resistant *Cynoglossus semilaevis* (DR-CS, family 14L006) and disease-susceptible *C. semilaevis* (DS-CS, family 14L104) against *Vibrio harveyi* infection. The results showed that following selective breeding, DR-CS had higher DNA methylation levels and different DNA methylation patterns, with 3,311 differentially methylated regions and 6,456 differentially methylated genes. Combining these data with the corresponding transcriptome data, we identified several immune-related genes that exhibited differential expression levels that were modulated by DNA methylation. Specifically, DNA methylation of tumor necrosis factor–like and lipopolysaccharide-binding protein-like was significantly correlated with their expression and significantly contributed to the disease resistance of the selected *C. semilaevis* family. In conclusion, we suggest that artificial selection for disease resistance in Chinese tongue sole causes changes in DNA methylation levels in important immune-related genes and that these epigenetic changes are potentially involved in multiple immune responses in Chinese tongue sole.

## Introduction

Epigenetic modifications, which are influenced by external environmental factors and predetermined inherited programs, induce changes in gene activity without altering the underlying DNA sequence (Jablonka and Lamb, 2002; Long et al., 2014). DNA methylation, the most widely and well-understood type of epigenetic modification, has been reported to play crucial roles in diverse processes such as X chromosome inactivation, embryogenesis, genomic imprinting, transposon silencing, and the onset of diseases (Yang et al., 2016). DNA methylation is performed by a set of enzymes called DNA methyltransferases, in which a methyl group (CH_3_) is added to position 5 of the pyrimidine ring of a cytosine (5 mC) (Bestor, 1990; Goll and Bestor, 2005). In most animals, the majority of methylated cytosines occur at CpG dinucleotides, whereas in plants and fungi, a large fraction of DNA methylation also occurs at CHG or CHH (where H = A, C, or T) (Feng et al., 2010; Junhyun et al., 2015).

In recent years, whole-genome analysis of DNA methylation has become an effective approach for researching human diseases (Zong et al., 2015) and will provide potential theoretical support and new targets for our study. DNA methylation is also increasingly recognized as prominent in diverse immune processes (Teitell and Richardson, 2003). Epigenetic changes, which mainly alter DNA methylation profiles, have been implicated in various types of cancers. Hypermethylation of CpG islands in the promoter region results in transcriptional silencing of tumor suppressor genes, whereas hypomethylation leads to oncogene activation in many cancers (Baylin and Jones, 2011; Esteller, 2011; Rodríguez-Paredes and Esteller, 2011; Chatterjee and Vinson, 2012). Methylation has important functions in tumor initiation and progression, and changes in methylation have been used as potential biomarkers for the early detection of cancers (Meng et al., 2012; Farkas et al., 2013; Guo et al., 2015; Vedeld et al., 2015).

In fish, genome-wide DNA methylation studies have been conducted to uncover the epigenetic effects on muscular polyunsaturated fatty acid metabolism of the common carp (*Cyprinus carpio*) (Zhang et al., 2019a), skin color variations in crucian carp [*Carassius carassius* L. (Zhang et al., 2017b) and *C. carpio* (Li et al., 2015)], behavioral effects of zebrafish (*Danio rerio*) (Olsvik et al., 2019), the evolution of heteromorphic sex chromosomes of three-spine stickleback (*Gasterosteus aculeatus*) (Metzger and Schulte, 2018), sexual dimorphism of hybrid tilapia (*Oreochromis* spp.) (Wan et al., 2016), sex determination of *C. semilaevis* (Shao et al., 2014), growth of large yellow croaker (*Larimichthys crocea*) (Zhang et al., 2019b), thermal acclimation of *G. aculeatus* (Metzger and Schulte, 2017), and so on. DNA methylation also functions in fish immune responses against diseases. For example, the loss of the regulator ubiquitin-like protein containing PHD and RING finger domains 1 (uhrf1) leads to tumor necrosis factor α (*TNF*-α) promoter hypomethylation and *TNF*-α activation. The changes in *TNF*-α expression promote cell shedding, a rapid loss of intestinal barrier function, and the recruitment of a series of immune cells (Marjoram et al., 2015). It has also been demonstrated that DNA methylation in *Ctenopharyngodon idella* was highly correlated with resistance against grass carp reovirus, probably due to the negative modulation of antiviral transcription (Shang et al., 2015; Shang et al., 2016).

Chinese tongue sole (*Cynoglossus semilaevis*) is a commercially valuable flatfish in China. However, the development of *C. semilaevis* aquaculture has been severely threatened by the outbreak of several bacterial- and viral-related diseases (Zhang et al., 2015). Fortunately, the publication of the article on the genome of *C. semilaevis* has laid a very important foundation for genome-wide methylation research (Chen et al., 2014). In previous studies, disease-resistant families against *Vibrio harveyi* infections were developed and bred. Subsequent challenge experiments found these fish had significantly higher survival (Chen et al., 2010). It was verified that different *C. semilaevis* families (resistant families vs. nonresistant families) showed obvious genetic variations in MHC IIB, which is a candidate molecular marker for resistance/susceptibility to various diseases (Niu et al., 2015). Further study is required to establish whether fish immunity is influenced by DNA methylation and to determine which genes have a role in this process. Here, we addressed these questions using a comprehensive analysis of the whole-genome DNA methylome and transcriptome of Chinese tongue sole immune tissues (liver, spleen, and kidney), which were compared between disease-resistant and disease-susceptible families. Our study revealed that significant differences exist between these two families and that DNA (de)methylation processes may play critical roles in certain immune response pathways.

## Materials and Methods

### Ethics Statement

The collection and handling of the animals in the study was approved by the Chinese Academy of Fishery Sciences’ animal care and use committee, and all experimental animal protocols were carried out in accordance with the guidelines for the care and use of laboratory animals at the Chinese Academy of Fishery Sciences.

### Sample Collection

Disease-resistant and disease-susceptible families of Chinese tongue sole were established by our research group from 2014 to 2015. The family establishment method is described in Chen et al. (2010). Briefly, cultured male and female populations were used as basic populations. All of the established families (1 year old) were chosen for challenge experiments against *V. harveyi*, which showed that the 14L006 family had strong disease resistance, with a final survival rate of 93.46%; the 14L104 family had low disease resistance, with a final survival rate of 9.15%. We also assessed some growth-related traits, and the *T* test showed no significant differences between the 14L006 and 14L104 families in body weight (*P* = 0.140) or length (*P* = 0.704). For each family (14L006 and 14L104), three nonchallenged fish were anesthetized by using MS-222. Then, three immune-related tissues, including the liver, spleen, and kidney of each fish, were isolated and stored at −80°C until DNA or RNA extraction. The experimental fish were approximately 1.5 years old with an average length of 22.7 ± 3.2 cm and an average weight of 81.8 ± 5.2 g. The fish were acclimatized for 7 days before the experiments. The animals were collected and handled in accordance with the guidelines for the care and use of laboratory animals at the Chinese Academy of Fishery Sciences.

### Whole-Genome Bisulfite Sequencing

Genomic DNA was extracted from each tissue using a DNeasy Blood & Tissue Kit (Qiagen GmbH, Hilden, Germany) according to the manufacturer’s recommendations. DNA purity was monitored on agarose gels. The DNA from nine immune-related tissues of the same family was pooled equally. DNA libraries were prepared following a previously described method (Zhang et al., 2017a). For library construction, a total of 5.2 µg mixed DNA spiked with 26 ng lambda DNA was fragmented into 200 to 300 bp by sonication, followed by terminal repairing and adenylation–ligation. Then, sonicated DNAs from different samples were ligated with different cytosine-methylated barcodes. The DNA fragments were treated twice with bisulfite using the EZ DNA Methylation-Gold^™^ Kit (Zymo Research), and the resulting single-strand DNA fragments were amplified using the KAPA HiFi HotStart Uracil + ReadyMix (2X) (Kapa Biosystems, Wilmington, MA, USA). The concentration of the library was quantified by using a Qubit^®^ 2.0 Fluorometer. Then, an Agilent Bioanalyzer 2100 system was applied to assess the insert size. The barcode-ligated samples were clustered by a cBot Cluster Generation System *via* TruSeq PE Cluster Kit v3-cBot-HS, followed by sequencing on an Illumina HiSeq 2500 platform (Novogene Bioinformatics Institute, Beijing, China). Finally, 100-bp paired-end reads were generated after image analysis and base calling with the standard Illumina pipeline.

### Data Analysis

Read sequences produced by the Illumina pipeline in FASTQ format were first preprocessed using in-house Perl scripts with the following steps: (1) remove reads with adaptor; (2) remove reads with the percentage of N (unknown bases) larger than 10%; and (3) remove reads with low quality (PHRED ≤5, percentage of low-quality bases ≥50%). All subsequent analyses were based on clean reads. The remaining reads that passed the filters were called clean reads, and all of the subsequent analyses were based on them. The clean reads and reference genome were transformed into bisulfite-converted sequences (C-to-T and G-to-A converted). Then, Bismark software (0.16.3) (Krueger and Andrews, 2011) and the aligner engine of bowtie2 (2.2.5) (Langmead and Salzberg, 2012) were used to perform the alignment of the converted clean reads to the *C. semilaevis* reference genome with the following set of parameters: –score_min L, 0, -0.2, -X 700 –dovetail. The clean reads that produce a unique best alignment from the two alignment processes (original top and bottom strand) were then compared to the normal genomic sequence, and the methylation state of all cytosine positions in the read was inferred by Bismark (bismark_methylation_extractor) with the parameters –multicore 4 –paired-end –no_overlap -ignore 5 –ignore_r2 5. The sequencing depth and coverage were summarized using deduplicated reads performed by Bismark (deduplicate_bismark) with parameters –paired –samtools_path. The methylation extractor results were transformed into bigWig format for visualization using the IGV browser. The sodium bisulfite nonconversion rate was calculated as the percentage of cytosines sequenced at cytosine reference positions in the lambda genome.

A window size *w* = 3,000 bp and step size of 600 bp (Smallwood et al., 2014) were selected, and the sum of the methylated and unmethylated read counts in each window was calculated. The methylation level (ML) for each CpG site shows the fraction of methylated Cs (mC) and is defined by the following equation: ML = reads(mC)/reads(mC+umC), where umC are the nonmethylated Cs. The calculated ML was further corrected with the bisulfite nonconversion rate as described (Lister et al., 2013).

### Differentially Methylated Region Analysis

Differentially methylated regions (DMRs) were identified using the swDMR software (https://sourceforge.net/projects/swdmr/), which uses a sliding-window approach. The window was set to 1,000 bp and step length to 100 bp, and only windows with at least 10 informative CpGs were considered. Fisher exact test was used to detect the DMRs. Windows in which a greater than two-fold ML change was identified with an adjusted *P* < 0.05 were considered DMRs. Differentially methylated genes (DMGs) were defined as genes containing DMRs in any part of the gene features, where putative promoter regions were designated from −2 kb to the transcription start site (TSS). Fisher test was used to detect the DMRs.

To check the reliability of the whole-genome bisulfite sequencing (WGBS), DMRs located in genes such as gramd1b, KCNH4, plekhg5, TNF-like, and lipopolysaccharide (LPS)–binding protein-like (LBP-like) were selected for verification of the WGBS data by bisulfate polymerase chain reaction (BS-PCR) analysis for DNA methylation. DNA samples from nine immune-related tissues of the same family were pooled equally, and then the mixed DNA was sodium bisulfite modified using a kit following the manufacturer’s instructions. Amplification primers for BS-PCR were designed using MethPrimer design software (http://www.urogene.org/methprimer/) (**Supplementary Table S1**). Polymerase chain reaction was performed with TaKaRa EpiTaq HS (Takara, Japan) following the manufacturer’s instructions. The PCR was performed in a volume of 25 µL, containing 2.5 µL 10× EpiTaq PCR buffer, 2.5 µL MgCl_2_ (25 mM), 3 µL of dNTP Mixture (2.5 mM for each dNTP), 1 µL of each forward and reverse primer (10 µM), 0.15 µL EpiTaq HS DNA Polymerase, 1 µL bisulfite-treated genomic DNA, and 13.85 µL ddH_2_O. The PCR amplification conditions were as follows: denaturation at 98℃ for 3 min, then 35 cycles of 98℃ for 10 s, 55 for 30 s, and 72° Cfor 30 s, and a final extension at 72°C for 7 min. The amplified products were purified and cloned into the pEASY-T1 vector, and at least 10 clones per fish, tissue, and family were randomly selected for sequencing.

### Rna-Seq

Total RNA from liver, spleen, and kidney samples was extracted using an EasyPure RNA Kit (TransGen, Beijing, China) according to the manufacturer’s instructions. The integrity and quality of the total RNA were determined using an Agilent 2100 NanoDrop and agarose gel electrophoresis. For the RNA-seq libraries, RNA from nine immune-related tissues of the same family was pooled equally. A total of 3 µg RNA per family was used as input material for the RNA sample. Sequencing libraries were generated using the NEBNext^®^ Ultra^™^ RNA Library Prep Kit for Illumina^®^ (NEB, USA) following the manufacturer’s recommendations. Fragments per kilobase of exon per million fragments (FPKM) of each gene were calculated based on the length of the gene and read count mapped to this gene. Prior to differential gene expression analysis, for each sequenced library, the read counts were adjusted by the edgeR program package through one scaling-normalized factor. Differential expression analysis of two conditions was performed using the DEGSeq R package (1.20.0). Transcripts with a *P* < 0.05 were assigned as significantly differentially expressed.

To check the reliability of the RNA-seq results, some of the differentially expressed genes (DEGs) were randomly selected for quantitative reverse transcriptase (RT)–PCR verification, including thsd7b, plce1, ddr2, c7h6orf58, TNF-like, and LBP-like. The amplification primers for qRT-PCR are shown in **Supplementary Table S1**. RNA from nine immune-related tissues of the same family was pooled equally, and then cDNA was synthesized with PrimeScript^™^ II 1st Strand cDNA Synthesis Kit (Takara). The qRT-PCR was performed using SYBR^®^ Premix Ex Taq TM Ⅱ (Tli RNase H Plus) according to the manufacturer’s instructions. The PCR program was 9°C or 30 s, followed by 40 cycles of 95°C for 5 s and 60°C for 30 s. All samples were run thrice. The relative expression levels were calculated according to the 2^−△△Ct^ method. Statistical significance was determined by one-way analysis of variance. The significance was set at *P* < 0.05.

### Luciferase Reporter Assay

Lipopolysaccharide-binding protein-like promoter regions (−3056 to −1657) containing DMRs were amplified and cloned into the pGL3-basic vector (**Supplementary Table S1**). Whole reporter-gene plasmids were methylated *in vitro* using *M.Sss*I methylase (New England BioLabs) following the manufacturer’s protocol. Successful vector methylation was checked by analyzing the band patterns by gel electrophoresis after digestion of the purified plasmids with the *Hpa*II enzyme, which digests only unmethylated DNA.

HEK293T cells were cultured in Dulbecco modified Eagle medium (DMEM) supplemented with 10% (vol/vol) heat-inactivated fetal bovine serum and antibiotics (100 IU/mL penicillin and 100 µg/mL streptomycin). HEK293T cells were grown at 37°C and supplied with 5% CO_2_. Following overnight culturing in 24-well plates, the cells were transfected using Lipofectamine 2000 (Invitrogen). For transfections, 400 ng/well of methylated or unmethylated plasmids was used, and 40 ng/well pRL-TK plasmid served as an internal control. After 48 h, cells were lysed, and luciferase activity was measured with a dual luciferase report gene assay kit (Beyotime) following the manufacturer’s instructions. The assay was repeated three times.

## Results

### Disease-Associated Methylation Profiles

**Figure 1:**
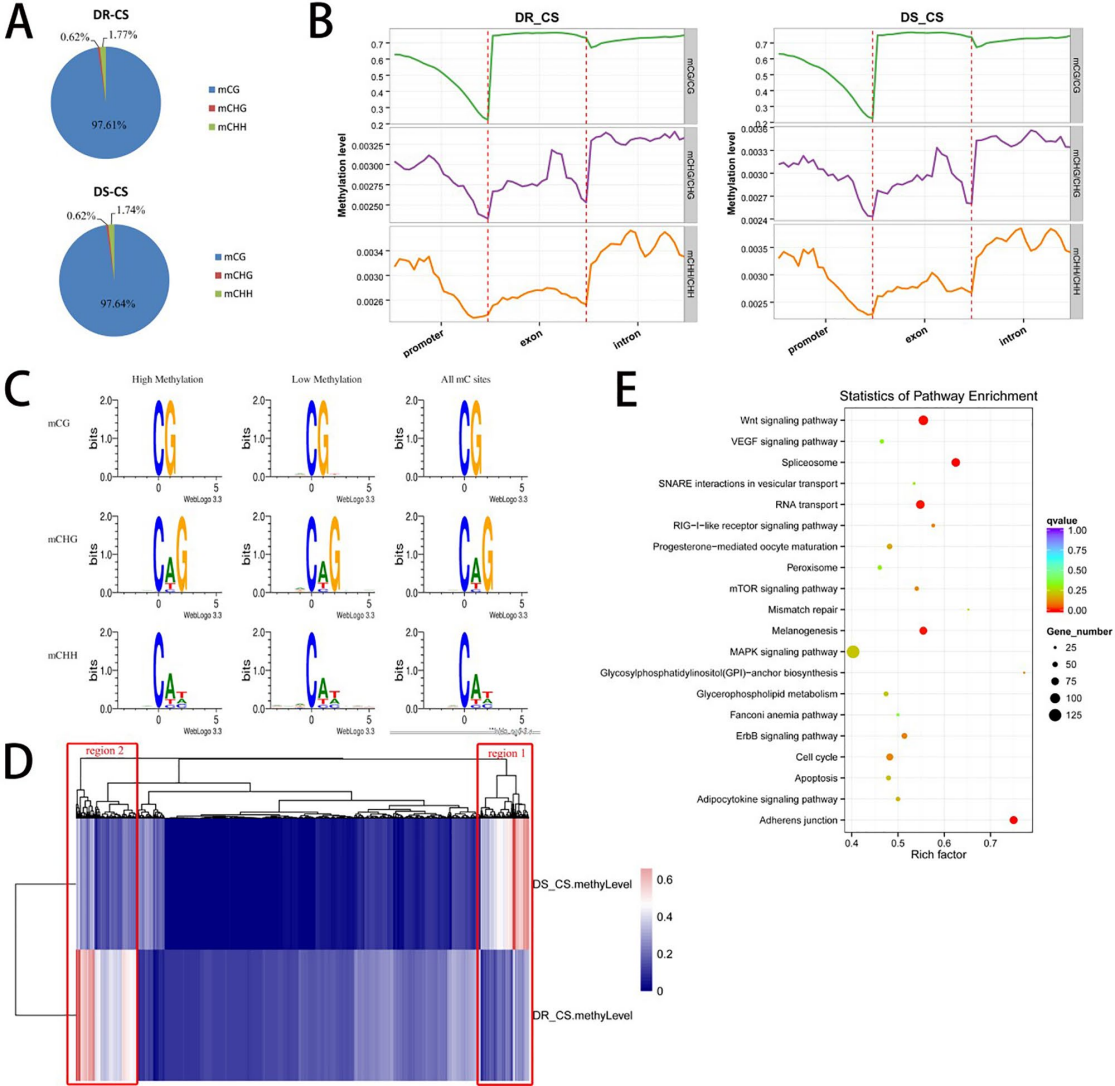
DNA methylation of the DR-CS and DS-CS families. **(A)** Percentage of mCs in the mCG, mCHG, and mCHH contexts. **(B)** DNA methylation levels in functional regions of the genome. The gene features include the promoter (the 2 kb region upstream of the transcription start site [TSS]), exons, and introns. The ordinate indicates the methylation level. **(C)** mCG, mCHG, and mCHH sequence motifs. The abscissa indicates the base positions. Bits indicate the occurrence of nucleotides. Four different colors represent the bases. **(D)** Cluster analysis of differentially methylated regions (DMRs) using a heat map. Red represents hypermethylation, and blue represents hypomethylation. The two regions with significant differences are marked in red boxes. **(E)** KEGG analysis of nonredundant DMR-associated genes. The *x* axis is the rich factor, and the *y* axis is the KEGG pathway classification.

To obtain the DNA MLs of disease-resistant (DR-CS) and disease-susceptible (DS-CR) families at base-pair resolution, we performed WGBS. For DR-CS and DS-CS, 15.52 and 13.36 Gb of clean bases were produced, with 78.39% and 72.78%, respectively, of genomic cytosines (Cs) being covered by at least five unique reads (**Supplementary Table S2**). These data were deposited in the NCBI SRA database with the accession numbers SRR8447884 and SRR8447885. Among the detected methylation sites, the mC percentage of reference genomic cytosines was 4.44% and 3.38% for DR-CS and DS-CS, respectively; mCG sites accounted for 43.12% and 32.82% of DR-CS and DS-CS; and mCHG and mCHH sites accounted for 0.12% and 0.09% of DR-CS and DS-CS (**Supplementary Table S3**). Among the mC sites we identified, more than 97% were in the mCG dinucleotide context, while non-CG MLs were very low (mCHG 0.6% and mCHH 1.7%) (**Supplementary Table S4**; **Figure 1A**). The methylation status of mCGs in various genomic elements was analyzed, showing that mCGs had slightly lower DNA MLs in promoter elements than in exons and introns; however, mCHG and mCHH MLs were higher in introns (**Figure 1B**; **Supplementary Figure S1**). To obtain an overview of the detected DNA methylation, we examined chromosome-wide MLs, which consists of 20 euchromatin and 2 heterochromatin. Circos analysis visualizing data and information in a circular layout showed that DR-CS and DS-CS had relatively similar mC levels and mC densities in the same chromosome (**Supplementary Figure S2**). A relatively high methylation density of the W chromosome (NC_024327.1) in both DR-CS and DS-CS was identified, which was probably associated with its high repeat content (**Supplementary Figure S3**). The site preference of CHG and CHH was analyzed, and logo plots showed that they were highly correlated with sequence context, with CAG/CTG the most frequent CHG (**Figure 1C**).

### DMR Between DR-CS and DS-CS

A total of 3,311 DMRs were detected between the two families, including 2,959 hyper-DMRs (hypermethylation in DR-CS compared with DS-CS) and 352 hypo-DMRs (hypomethylation in DR-CS compared with DS-CS) (*P* < 0.05, **Supplementary Table S5**). We identified 6,456 DMGs that harbored DMRs in their promoter, exon, or intron regions, including 4,504 DMGs containing DMRs in their promoter regions (**Supplementary Table S5**). Gene ontology (GO) and directed acyclic graph (DAG) enrichment analyses showed that DMGs were overrepresented for “positive regulation of mating type–specific transcription DNA-templated” among the following biological processes: nitrogen compound metabolic process, gene expression, cellular nitrogen compound metabolic process, cellular aromatic compound metabolic process, nucleobase-containing compound metabolic process, RNA metabolic process, and organic cyclic compound metabolic process (**Supplementary Figure S4**). Differentially methylated genes were also enriched in nucleic acid binding under molecular function, including heterocyclic compound binding and organic cyclic compound binding (**Supplementary Figure S5**). This pattern appears to be related to the regulation of transcription, as positive regulation of mating type–specific transcription and nucleic acid binding are both part of the transcription process.

Cluster analysis of the DMRs was conducted using a heat map, in which two significantly different regions (region1 and region2) were found between the DR-CS and DS-CS families (**Figure 1D**). Gene ontology enrichment analysis showed that region1 was clustered into cellular response to stimulus and response to stimulus in biological process (**Supplementary Figure S6**), while region2 was clustered into interleukin 4 (IL-4) receptor binding and growth factor receptor binding (**Supplementary Figure S7**). KEGG pathway analysis identified four significantly enriched (*P* < 0.05) and immune-related biological pathways, including the RIG-I–like receptor signaling pathway, mTOR signaling pathway, apoptosis, and MAPK signaling pathway (**Figure 1E**).

To further validate the technical reproducibility of our results, we randomly selected three genes that harbored DMRs (gramd1b, KCNH4, and plekhg5) to perform bisulfite sequencing experiments on the same samples. We observed good consistency between the WGBS and bisulfite sequencing results by Pearson correlation analysis (*r* = 1.000, *P* = 0.002) (**Supplementary Table S1**, **Supplementary Figure S8**).

### Correlations Between Methylation and Gene Expression

**Figure 2:**
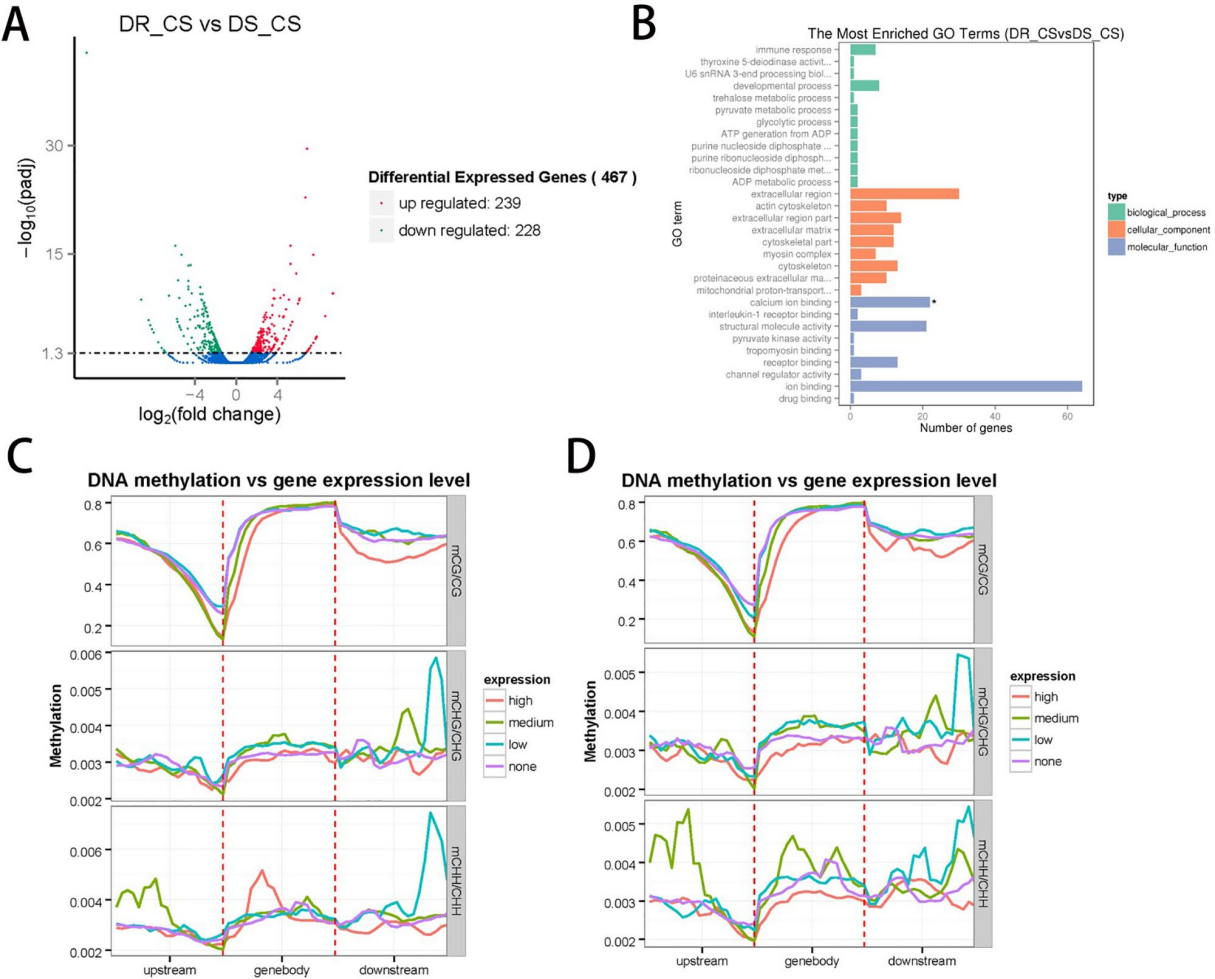
Integrated analysis of the genome-wide DNA methylation and gene expression profiles. **(A)** The number of differentially expressed genes (DEGs) identified by comparing DR-CS and DS-CS. The red dots represent the 239 upregulated genes in DR-CS compared with DS-CS. The green dots represent the 228 downregulated genes in DR-CS compared with DS-CS. **(B)** Gene ontology enrichment analysis of the downregulated DEGs. The GO enrichment analysis results for the differentially expressed genes are classified into the following three categories: biological process (green histogram), cellular component (orange histogram), and molecular function (blue histogram). The *x* axis is the corresponding number of genes, and the *y* axis is the gene ontology (GO) gene function classification. * indicates significant enrichment (*P* adjusted value < 0.05). **(C)** Methylation levels in CG, CHG, and CHH contexts of differentially expressed genes from DR-CS. The DEGs were divided into the following four groups according to their expression levels: none (FPKM <1), low (1 < FPKM < Q1), medium (Q1 < FPKM < Q3), high (FPKM > Q3). **(D)** Methylation levels in CG, CHG, and CHH contexts of differentially expressed genes from DS-CS. The DEGs were divided into the following four groups according to their expression level: none (FPKM < 1), low (1 < FPKM < Q1), medium (Q1 < FPKM < Q3), high (FPKM > Q3).

To identify the DEGs between the two families and assess the relationship between DNA methylation and gene expression, we measured gene expression profiles by RNA sequencing using the same tissues used for the DNA methylation studies. A total of 117,880,006 (59,143,966 in DR-CS and 58,736,040 in DS-CS) clean reads with a Q20 percentage of 96% were generated and used for the subsequent analysis. In total, transcripts from 467 DEGs were identified, with 239 of them being significantly upregulated and 228 of them being downregulated in the DR-CS compared with the DS-CS family (**Figure 2A**). To assign functional information to the transcripts, upregulated or downregulated DEGs were selected for annotation. Gene ontology annotation indicated that seven downregulated DEGs were annotated to immune response (GO:0006955) (**Figure 2B**), which may provide an explanation for the changes experienced in the key components of the defense mechanism. KEGG pathway analysis identified two significantly enriched (*P* < 0.05) and immune-related biological pathways, including the NOD-like receptor signaling pathway and Toll-like receptor signaling pathway. The transcriptome data were deposited in the NCBI SRA database with accession numbers SRR9009084 and SRR9009085.

To further validate the technical reproducibility of our results, we randomly selected four DEGs (thsd7b, plce1, ddr2, and c7h6orf58) to perform qRT-PCR experiments on the same samples. Most of the qRT-PCR results were consistent with the transcriptome data, and Pearson correlation analysis identified that RNA-seq showed a moderate correlation with qRT-PCR (*r* = 0.447, *P* = 0.553) (**Supplementary Table S1**, **Supplementary Figure S9**).

To examine the relationships between expression and methylation, genes were sorted into four groups according to their expression levels. We found a general trend of negative associations between expression levels and MLs for CG contexts, but there was no distinct trend for the CHH and CHG contexts (**Figures 2C, D**). By comparing the lists of DMGs and DEGs, we identified 59 DEGs with statistically significant methylation variations, including 52 hypermethylated DMRs (DR-CS compared with DS-CS) and 7 hypomethylated DMRs (DR-CS compared with DS-CS). KEGG analysis found three immune-related genes, including TNF-like (XM_008324037.1), dual specificity phosphatase 2 (dusp2, XM_008336172.1), and Toll-like receptor 5 (TLR5, XM_008313329.1), which participate in the Toll-like receptor signaling pathway, NOD-like receptor signaling pathway, RIG-I–like receptor signaling pathway, mTOR signaling pathway, MAPK signaling pathway, adipocytokine signaling pathway, transforming growth factor β signaling pathway, apoptosis, cytokine–cytokine receptor interaction, and herpes simplex infection.

### TNF-Like as an Epigenetic Target Contributes to Disease Resistance

**Figure 3:**
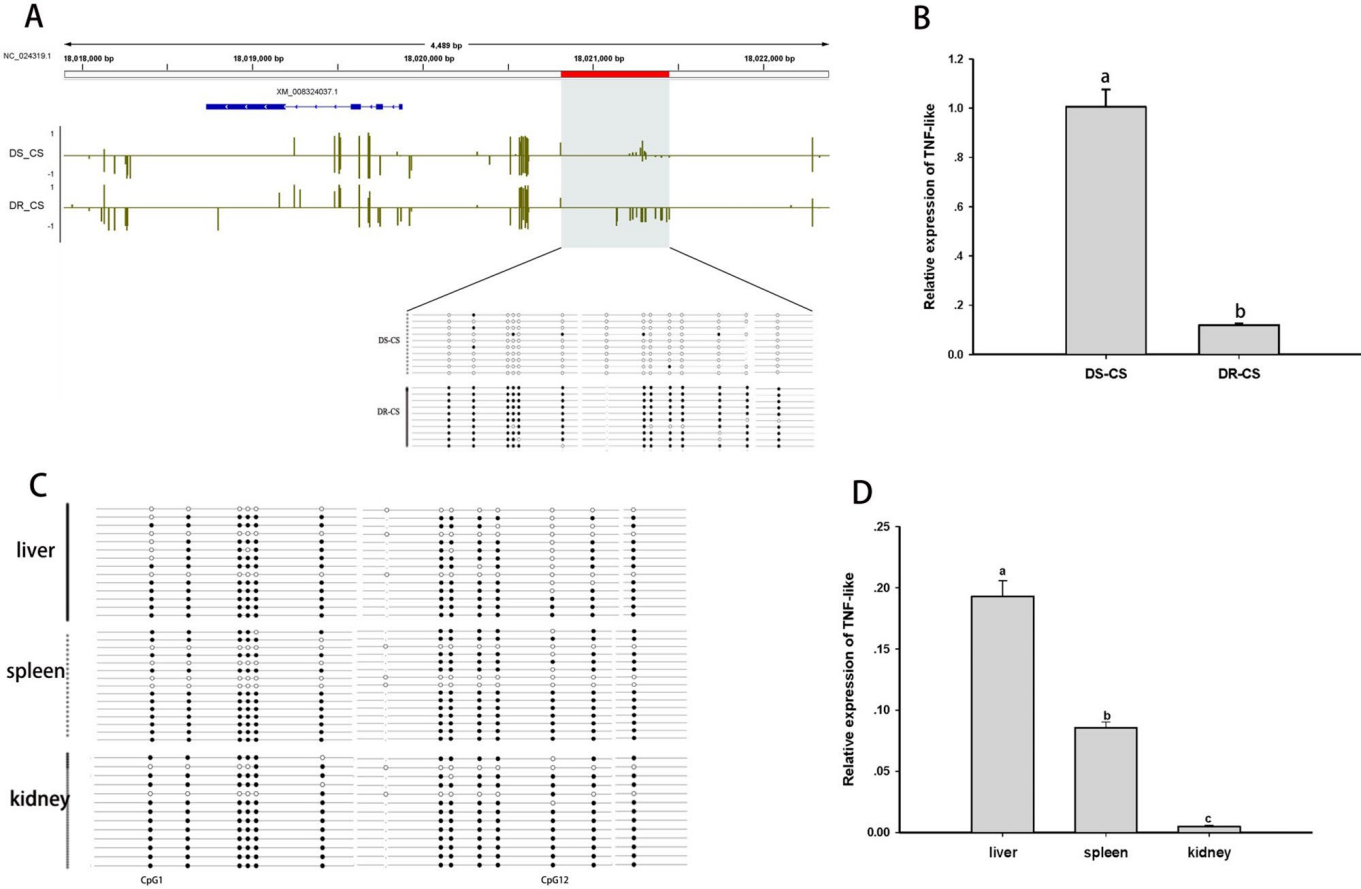
Differential methylation and expression of the tumor necrosis factor (TNF)–like gene. **(A)** DNA methylation profiles for the TNF-like gene in the DS-CS and DR-CS families. The gene structures are shown at the top of the graph, with blue boxes representing exons and arrows indicating introns. The shaded regions represent DMRs. Yellow vertical lines indicate the methylation level of cytosines identified by WGBS. The DMRs were confirmed by BS-PCR, and the filled or open circles indicate methylated or unmethylated CpG sites, respectively. Each row represents one sequenced clone. **(B)** The relative expression of TNF-like in the DR-CS and DS-CS immune tissues. Different letters a and b indicate significant differences (*P* < 0.05). **(C)** DNA methylation profiles for tumor necrosis factor (TNF)–like in the liver, spleen and kidney from DR-CS. The filled or open circles indicate methylated or unmethylated CpG sites, respectively, and each row represents one sequenced clone. **(D)** The relative expression of TNF-like in the liver, spleen, and kidney from DR-CS. Different letters a, b, and c indicate significant differences (*P* < 0.05).

The TNF-like gene was selected for further analysis of the relationship between aberrant DNA methylation and mRNA transcription. Based on BS-PCR analysis, significant differences in DNA methylation were confirmed between the two families (*P* < 0.05) (**Figure 3A**). Furthermore, the DR-CS family showed significantly higher DNA MLs compared with the DS-CS family. The results indicated that 13 of 14 CpG sites present in the TNF-like gene DMR region had significantly higher MLs in DR-CS compared to the DS-CR family (*P* < 0.05). The TNF-like mRNA levels were quantified by qRT-PCR in matched tissue samples, and there was significantly higher expression in DS-CS than in the DR-CS family (*P* < 0.05, **Figure 3B**).

To further determine whether methylation changes in TNF-like affected gene expression, the correlations between DNA methylation and gene expression levels in different tissues (liver, spleen, and kidney) from DR-CS were analyzed. Methylation patterns for these 14 CpG sites for each tissue are shown in **Figure 3C**. The MLs of the CpG1 and CpG12 sites were essentially uniform across the tissues. The highest expression of TNF-like was observed in the liver, with significantly lower levels in the spleen (*P* < 0.05) and the lowest levels in the kidney (*P* < 0.05) (**Figure 3D**). The correlation analysis showed that there was a significant negative correlation between TNF-like MLs and mRNA expression (Pearson *r* = −0.997, *P* < 0.05). Specifically, the MLs of the CpG1 site (Pearson *r* = −0.995, *P* < 0.05) and CpG12 site (Pearson *r* = −0.996, *P* < 0.05) were significantly negatively correlated with mRNA expression.

### LBP-Like as an Epigenetic Target Contributes to Disease Resistance

**Figure 4:**
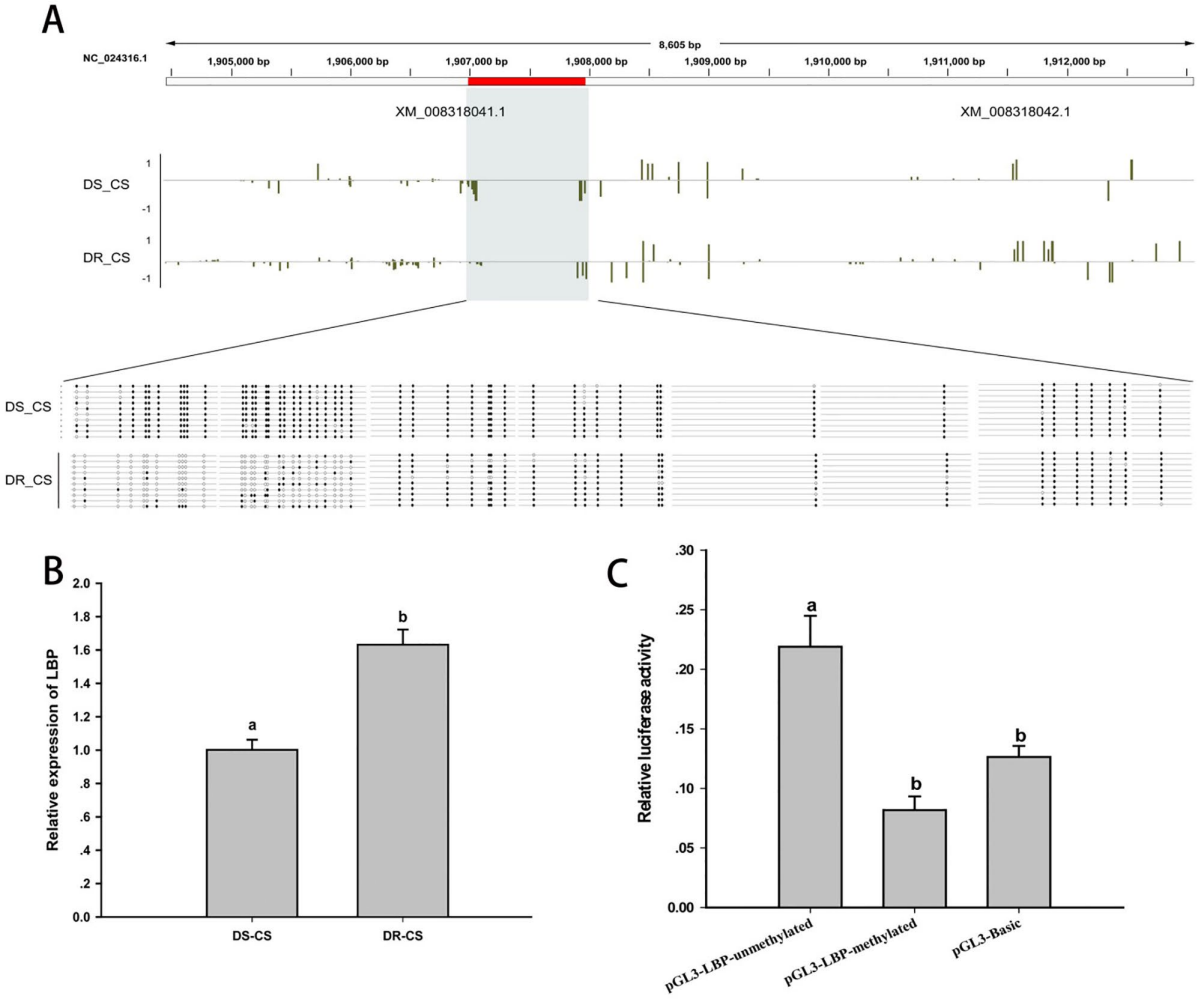
DNA methylation of lipopolysaccharide-binding protein-like (LBP-like) regulates gene expression. **(A)** DNA methylation profiles for the LBP-like gene in DS-CS and DR-CS. The shaded regions represent DMRs. Yellow vertical lines indicate the methylation level of cytosines identified by WGBS. The DMRs were confirmed by BS-PCR. Filled or open circles indicate methylated or unmethylated CpG sites, respectively, and each row represents one sequenced clone. **(B)** The relative expression of LBP-like in the immune tissues from DR-CS and DS-CS. Different letters a and b indicate significant differences (*P* < 0.05). **(C)** Luciferase assays for methylated or unmethylated recombinant plasmids. The *x* axis represents different recombinant plasmids, and the *y* axis represents the relative luciferase activity. Different letters a and b indicate significant differences (*P* < 0.05).

Differences in DNA MLs between DR-CS and DS-CS for the LBP-like DMR were of similar magnitude as observed in the WGBS results. By alignment and statistical analysis, MLs of the CpG sites located at 1906984–1907244 were found to be significantly higher in the DS-CS family than in the DR-CS family (*P* < 0.05). All of the former 27 CpG sites except CpG2 and CpG23 possessed highly significant discrepancies in MLs between the DR-CS and DS-CS families (*P* < 0.05) (**Figure 4A**).

To further assess whether the methylation of the CpG loci could act as regulatory elements for gene expression, LBP-like mRNA expression from the DR-CS and DS-CS families was examined by qRT-PCR. Although the results were different from the results obtained with RNA-seq, qRT-PCR showed that the average mRNA expression of LBP-like was significantly higher in the DR-CS family (*P* < 0.05, **Figure 4B**), indicating a negative correlation with CpG loci ML.

To study the effects of DNA methylation on LBP-like promoter activity, pGL3-LBP plasmids were methylated by *M. Sss*I methylase *in vitro*. Subsequently, the methylated or unmethylated plasmids were transfected into HEK293T cells, and promoter activity was compared with the negative control (pGL3-Basic plasmid). The results indicated that methylation of the pGL3-LBP plasmids led to a significant repression of promoter activity (*P* < 0.05) (**Figure 4C**).

## Discussion

In this study, we provide the first comprehensive investigation of the DNA methylation and transcriptome relationships underlying the differences between *V. harveyi*-resistant and -susceptible Chinese tongue sole, thus providing insights into the role of epigenetics in the regulation of bony fish immunity. The two families used in this study were derived from a long-term selection, and there may be significant differences in other phenotypic traits besides the disease resistance trait, such as fatty acid metabolism, skin color variations, thermal acclimation, and sex ratio. Although in this research we focused on the relationship between methylation and disease resistance, we cannot deny that differences in DNA methylation between the two families may also be related to other traits.

### DNA Methylation Patterns

Bacterial challenge experiments demonstrated that there were significant differences in survival of *V. harveyi* infection between the DR-CS and DS-CS families. In this study, WGBS was used to explore whether there were differences in DNA methylation between these two families. The results showed that the mC percentage of DR-CS was higher than in the DS-CS family, and the mCG percentage of DR-CS was higher than in the DS-CS family. Therefore, it is proven that DNA MLs were correspondingly modified during the selected breeding of *C. semilaevis*, implying that DNA methylation may play important roles in regulating functional gene expression associated with resistance traits. Similarly, in the selected breeding of scallop (*Patinopecten yessoensis*), the rate of methylation of polymorphic fragments in “Yubei” was higher than that in the control group (Wu et al., 2015). Genomic DNA MLs are altered by cold stress and inherited across multiple generations in Nile tilapia (Zhu et al., 2013). The average MLs of mCGs (43.12% and 32.82%) in this study were lower than in maternal zebrafish (70%–95%) (Potok et al., 2013) or gonads from *C. semilaevis* (86% average) (Shao et al., 2014). Differences in the MLs of these species may be attributed to the different materials used in the experiments.

Next, the methylation patterns in various functional elements, such as the promoter, exon, and intron, were checked. The results showed that the unmethylated CpG sites were highly enriched in the promoter regions and that DNA MLs were dramatically decreased at the TSS (**Figure 1B**). The pattern of DNA methylation was similar to that observed in zebrafish (Jiang et al., 2013; Potok et al., 2013) and mammals (Molaro et al., 2011). This result is consistent with the “CG content rule” that regions with a high CpG ratio of observed over expected (obs/exp) are unmethylated (Potok et al., 2013). mCHG and mCHH MLs also decreased significantly toward the TSS, which occurs in human embryonic stem cells (Lister et al., 2009). We analyzed the surrounding DNA motifs for their CHG and CHH methylation patterns; the results showed that CHG MLs were higher than CHH levels, and we found more in CAG than in CTG (**Figure 1C**) contexts, which is similar to what has been observed in humans (Lister et al., 2009).

Using WGBS, 3311 regions were identified with significant methylation differences between the DR-CS and DS-CS families. In total, 65% of the DMRs were found within the promoter regions, while 56% and 61% of the DMRs were found within the exon and intron regions, respectively. Overall, these DMRs were widely distributed along the genome. It is widely accepted that DNA methylation has active roles in gene regulation (Li et al., 1992; Li et al., 1993; Shao et al., 2014; Sallustio et al., 2019). Gene ontology and DAG enrichment analysis implied that DNA methylation is involved in the transcription process by regulating a series of biological processes. We observed a remarkable methylation contrast in the immune-related biological pathways between the DR-CS and DS-CS families. In general, our study suggested that the genome-wide methylation patterns in *C. semilaevis* changed after selective breeding in the generation of the DR-CS families.

### Different Transcription Patterns

KEGG pathway analysis identified two significantly enriched (*P* < 0.05) biological pathways related to the immune systems, including the NOD-like receptor signaling pathway and the Toll-like receptor signaling pathway. Interestingly, most of the DEGs in the abovementioned signaling pathways had lower expression levels in the DR-CS compared to the DS-CS family, including TNF-α, IL-1β, IL-6, IL-12 and IL-8. Following a previous study in *C. semilaevis*, we compared the HOSG group, which shows obvious symptoms of infection, versus the NOSG group, which shows no obvious symptoms of infection. After challenge with *Vibrio anguillarum*, genes related to the NOD-like receptor signaling and the Toll-like receptor signaling pathways showed differential expression. In the HOSG group, several acute-phase proteins, such as IL-6, IL-1β, ferritin, and HSPs, were significantly upregulated (Zhang et al., 2015). The systemic immune response induced by noninfectious agents is called the systemic inflammatory response syndrome (SIRS), and the infection-induced systemic immune response is called sepsis. The host inflammatory responses are similar between SIRS and sepsis and may lead to multiple-organ dysfunction syndrome and ultimately death (Castellheim et al., 2009). We speculate that excessive inflammatory factors in the DS-CS family may affect organ function and contribute to their lower survival rate.

### Gene Expression Is Related to DNA Methylation

According to previous reports, hypermethylation at the promoter is often associated with gene repression (Li and Zhang, 2014), and the methylation location in intragenic regions is often influenced by the active expression of nearby genes and the regulation of alternative splice variants (Park et al., 2011; Farkas et al., 2013). However, not all genes conform to these rules (Zhang et al., 2017a). Interestingly, in some conditions, such as in human dendritic cells, gene activation precedes DNA demethylation in response to infection (Pacis et al., 2019). In our study, highly expressed genes correlated with lower DNA MLs in different genomic features, and a general trend of negative associations between expression and MLs for CG contexts was established (**Figure 2C, D**).

We identified 59 DEGs with statistically significant methylation variations, including 35 highly expressed genes (DR-CS compared with DS-CS) and 24 genes with lower expression (DR-CS compared with DS-CS), by comparing DMG and DEG gene lists. A close relationship was observed between these DMGs and disease resistance, and KEGG analysis found three immune-related genes. We selected the TNF-like and LBP-like genes for further research.

BLAST analysis showed that TNF-α has been characterized in several bony fish. It was found that TNF-α increases the susceptibility of zebrafish to viral (spring viremia of carp virus) and bacterial infections (*Streptococcus iniae*) (Roca et al., 2008). Similarly, TNF-α is poorly upregulated by immune challenge *in vitro* and *in vivo* in mammals (Laing et al., 2001; Garcíacastillo et al., 2002), and it weakly induces chemotaxis, respiratory burst, and phagocytosis and showed no response in macrophages (Zou et al., 2003; Grayfer et al., 2008). Based on these results, we speculated that the higher expression of TNF-like contributed to the susceptibility of DS-CS to *V. harveyi*. Furthermore, our correlation analysis between DNA methylation and gene expression of TNF-like showed that hypermethylation of the TNF-like promoter led to low expression of TNF-like mRNA. Previous research in zebrafish has demonstrated that epigenetic regulators, such as a ubiquitin-like protein containing PHD and RING finger domains 1 (uhrf1), reduce TNF-α promoter methylation in intestinal epithelial cells (IECs). Interestingly, the increased expression of TNF-α in IEC results in shedding and apoptosis, immune cell recruitment, and barrier dysfunction (Marjoram et al., 2015). These results suggest that TNF-α promoter methylation in DR-CS tends to be hypermethylated, coinciding with its downregulation, which may decrease the susceptibility of *C. semilaevis* to *V. harveyi* infections by protecting epithelial cells from damage. Additionally, the DNA methylation patterns of *C. semilaevis* were modified during the course of selective breeding to create the DR-CS family.

In our research, LBP-like cells showed hypomethylation and higher expression levels in DR-CS. Simultaneously, a luciferase reporter assay indicated that DNA methylation modification of the LBP-like promoter led to a significant repression of transcriptional activity (*P* < 0.05). The cDNA and amino acid sequence (accession no. XP_008316264) of *C. semilaevis* LBP-like has been identified, characterized, and named *C. semilaevis* bactericidal/permeability-increasing protein (*CsBPI*). It was found that recombinant CsBPI (rCsBPI) was able to bind to a number of Gram-negative bacteria, which leads to bacterial death through membrane permeabilization and structural destruction (Sun and Sun, 2016). Furthermore, rCsBPI can enhance the resistance of tongue sole against bacterial as well as viral infection (Sun and Sun, 2016). In mice, it was identified that LBP is essential for the rapid induction of an inflammatory response by small amounts of LPS or Gram-negative bacteria during the survival of intraperitoneal *Salmonella* infections (Jack et al., 1997). Recently, more experiments have indicated that LBP primarily acts as an LPS transporter to CD14 (Wright et al., 1990; Hailman et al., 1994; Schumann and Latz, 2000) and the Toll-like receptor complex (Kopp and Medzhitov, 1999; Means et al., 2000). In comparison, LBP-like showed higher expression in the DR-CS family, which suggests important roles in the immune response. Overall, we speculate that LBP-like DNA methylation is modified during selective breeding and mediates epigenetic regulatory mechanisms.

## Data Availability

This data was deposited in NCBI SRA database, the results of WGBS have accessed on NCBI, and the accession number is to SRR8447884 and SRR8447885. The transcriptome data was deposited in NCBI SRA database, with the accession number of SRR9009084 and SRR9009085.

## Author Contributions

SC obtained and designed the project. YX performed the experiments; YX and YZ wrote the manuscript; SC and YL instructed, organized and constructed disease-resistant and disease-susceptible families; TG and WX sampled the tissues; FP revised the manuscript; CS and SC designed the experiments and revised the MS.

## Funding

This work was supported by the National Nature Science Foundation (31530078, 31461163005), the Taishan Scholar Project Fund of Shandong, China, the Applied Basic Research Project of Qingdao City (16-5-1-52-jch), the Natural Science Foundation of Shandong Province (ZR2019BC009), the Advanced Talents Foundation of QAU (6651118016), the “First Class Fishery Discipline” programme in Shandong Province.

## Conflict of Interest Statement

The authors declare that the research was conducted in the absence of any commercial or financial relationships that could be construed as a potential conflict of interest.

## Supplementary Material

Supplementary Figure S1 DNA methylation levels of mCG, mCHG and mCHH in functional regions of the genome. The blue, green and red features represent the promoter (the 2 kb region upstream of the TSS), exon and intron functional regions, respectively.

Supplementary Figure S2 The circos plot representing the mC level, gene number density and mC density of each chromosome in **(A)** DR-CS and **(B)** DS-CS. The red, blue and purple lines represent the CG, CHG and CHH context, respectively.

Supplementary Figure S3 The circos plot representing the mC density of each chromosome in **(A)** DR-CS and **(B)** DS-CS. The mC density heat map from green to red indicates the density of methylation from low to high. The W chromosome number is NC_024327.1.

Supplementary Figure S4 DAG visualization of the hierarchical structure of the biological process. Children that represent a more specific instance of a parent term have an ‘is a’ relationship to the parent. The darker the color of the node, the higher its number of Blast hits and annotation score. All nodes contain the hit annotation scores in numbers.

Supplementary Figure S5 DAG visualization of the hierarchical structure of the molecular function. Children that represent a more specific instance of a parent term have an ‘is a’ relationship to the parent. The darker the color of the node, the higher its number of Blast hits and annotation score. All nodes contain the hit annotation scores in numbers.

Supplementary Figure S6 GO enrichment analysis of the DMGs from region 1. The GO enrichment analysis results for the differentially expressed genes were classified into the following three categories: biological process (BP), cellular component (CC) and molecular function (MF). The x-axis represents the GO gene function classification, and the y-axis represents the corresponding number of genes.

Supplementary Figure S7 GO enrichment analysis of the DMGs from region 2. The GO enrichment analysis results for the differentially expressed genes were classified into the following three categories: biological process (BP), cellular component (CC) and molecular function (MF). The x-axis represents the GO gene function classification, and the y-axis represents the corresponding number of genes.

Supplementary Figure S8 Sodium bisulfite clone sequencing results for the **(A)** gramd1b, **(B)** KCNH4 and **(C)** plekhg5 DMRs. Filled and open circles indicate methylated and unmethylated CpG sites, respectively, and each row represents one sequenced clone. At least 10 clones were sequenced for DR-CS or DS-CS.

Supplementary Figure S9 The relative expression of thsd7b, plce1, ddr2 and c7h6orf58 in the immune tissues from DR-CS (black bars) and DS-CS (gray bars). Asterisks indicate significant differences (*P*< 0.05) between DR-CS and DS-CS.

Supplementary Table S1 Primers used in this research.

Supplementary Table S3 Percentage of different contexts.

Supplementary Table S4 Percentage of mC in the mCG, mCHG, and mCHH contexts.

Supplementary Table S5 The DMR location and ID.
