# Supplementary material for: Differences in DNA Methylation Between Disease-Resistant and Disease-Susceptible Chinese Tongue Sole (Cynoglossus semilaevis) Families
Source: Front Genet. 2019 Sep 13;10:847. doi: 10.3389/fgene.2019.00847 (PMC6753864; doi:10.3389/fgene.2019.00847)
Supplement: Supplementary Figure S1 — DNA methylation levels of mCG, mCHG and mCHH in functional regions of the genome. The blue, green and red features represent the promoter (the 2 kb region upstream of the TSS), exon and intron functional regions, respectively. [file DataSheet_1.zip › Supplementary Table S1.docx]

**Table S1.** Primers used in this research.

| Gene  name | Primer  name | Sequence  (5’ – 3’) | Application |
| --- | --- | --- | --- |
| gramd1b | gramd1b-BSP-F | TATTTGTTATTTTAGTGTTTTTTTT | BSP |
|  | gramd1b-BSP-R | TAAACTCCACAAACCTCAACCTAAC |  |
| KCNH4 | kcnh4-BSP-F | TGTTGTTGATTGGATTATGGTTATAAT |  |
|  | kcnh4-BSP-R | ACAAATCCACAAATCTAATACTCAC |  |
| plekhg5 | plekhg5-BSP-F | GGGTTGTTTTAATATATTTTATTTGTTTTT |  |
|  | plekhg5-BSP-R | TTCTCATCACTCAACCTATCTAACC |  |
| TNF-like | TNF-like-BSP-F | AAATATTTGGGTAAGTTGAAAATAAT |  |
|  | TNF-like-BSP-R | TTAAAATACACCTCATCTCAAAATAC |  |
| LBP-like | LBP-like-BSP-F1 | AAAAATGTTTTTAAATGTTTAAAATTTTAG |  |
|  | LBP-like-BSP-R1 | TCCAATCCATCATACAATAAAACTC |  |
|  | LBP-like-BSP-F2 | TGTTTTTATTGGAGGGAAATATGTAG |  |
|  | LBP-like-BSP-R2 | AACCCATCAACAAAACAAAATAAAA |  |
| thsd7b | thsd7b-qRT-F | GGTCAGTGGGGTCAGTGCGT | qRT-PCR |
|  | thsd7b-qRT-R | CAGTGGGAGTGGTGGGTGG |  |
| plce1 | plce1-qRT-F | CGAACGGAGGTTGCGGCTACAT |  |
|  | plce1-qRT-R | GGTTGAGGGTGTTGCGGTGGAT |  |
| ddr2 | ddr2-qRT-F | CGGAGACGGAGATGGGGC |  |
|  | ddr2-qRT-R | TCACGTATCGCTGGGCGA |  |
| c7h6orf58 | c7h6orf58-qRT-F | TACTTGCTGGCAGACTGGTGCC |  |
|  | c7h6orf58-qRT-R | AGGAGGATGTGGCGTAGGTGGA |  |
| TNF-like | TNF-like-qRT-F | TTTTTCCTTCTCGCCCTTGC |  |
|  | TNF-like-qRT-R | GGTTTTGCTGCTGAACTGTTG |  |
| LBP-like | LBP-like-qRT-F | CAGAAAGTGAAGGGAATGAAGGC |  |
|  | LBP-like-qRT-R | CCCAGCACAAACAGGAGGAT |  |
| CsRpl13α | CsRpl13α-qRT-F | GTTTGCCCTCCTTGGTCG |  |
|  | CsRpl13α-qRT-R | TGCCTGCTTTGTCAGCTTGA |  |
| LBP-like | LBP-like-luci-F | CGGGGTACCCTGTTGGGCTGTGATACCG | luciferase |
|  | LBP-like-luci-R | CCCAAGCTTCAGGTGGAAGAGGCTTGGTA |  |
