# Supplementary material for: Differences in DNA Methylation Between Disease-Resistant and Disease-Susceptible Chinese Tongue Sole (Cynoglossus semilaevis) Families
Source: Front Genet. 2019 Sep 13;10:847. doi: 10.3389/fgene.2019.00847 (PMC6753864; doi:10.3389/fgene.2019.00847)
Supplement: Supplementary Figure S1 — DNA methylation levels of mCG, mCHG and mCHH in functional regions of the genome. The blue, green and red features represent the promoter (the 2 kb region upstream of the TSS), exon and intron functional regions, respectively. [file DataSheet_1.zip › Supplementary Table S2.docx]

**Table S2.** Statistic of WGBS-seq for each sample.

| Sample name | Clean reads | Clean bases  (G) | Unique  mapped  reads | Unique  mapped  ratio (%) | 1 × C  Coverage (%) | 5 × C  Coverage (%) | Error  Rate (%) |
| --- | --- | --- | --- | --- | --- | --- | --- |
| DR-CS | 124169112 | 15.52 | 42117232 | 67.84 | 89.71 | 78.39 | 0.01 |
| DS-CS | 106861170 | 13.36 | 31848605 | 59.61 | 89.05 | 72.78 | 0.01 |
