# Supplementary material for: Differences in DNA Methylation Between Disease-Resistant and Disease-Susceptible Chinese Tongue Sole (Cynoglossus semilaevis) Families
Source: Front Genet. 2019 Sep 13;10:847. doi: 10.3389/fgene.2019.00847 (PMC6753864; doi:10.3389/fgene.2019.00847)
Supplement: Supplementary Figure S1 — DNA methylation levels of mCG, mCHG and mCHH in functional regions of the genome. The blue, green and red features represent the promoter (the 2 kb region upstream of the TSS), exon and intron functional regions, respectively. [file DataSheet_1.zip › Supplementary Table S3.docx]

**Table S3.** Percentage of different context.

| Sample  Name | mC percent  (%) | mCpG percent (%) | mCHG percent (%) | mCHH percent (%) |
| --- | --- | --- | --- | --- |
| DR-CS | 4.44 | 43.12 | 0.12 | 0.12 |
| DS-CS | 3.38 | 32.82 | 0.09 | 0.09 |
