# Supplementary material for: Differences in DNA Methylation Between Disease-Resistant and Disease-Susceptible Chinese Tongue Sole (Cynoglossus semilaevis) Families
Source: Front Genet. 2019 Sep 13;10:847. doi: 10.3389/fgene.2019.00847 (PMC6753864; doi:10.3389/fgene.2019.00847)
Supplement: Supplementary Figure S1 — DNA methylation levels of mCG, mCHG and mCHH in functional regions of the genome. The blue, green and red features represent the promoter (the 2 kb region upstream of the TSS), exon and intron functional regions, respectively. [file DataSheet_1.zip › Supplementary Table S4.docx]

**Table S4.** Percentage of mC in the mCG, mCHG, and mCHH contexts.

| Sample  name | mC | mCG | mCHG | mCHH |
| --- | --- | --- | --- | --- |
| DR-CS | 8084092 (100%) | 7890740 (97.61%) | 50323 (0.62%) | 143029 (1.77%) |
| DS-CS | 6151537 (100%) | 6006422 (97.64%) | 38083 (0.62%) | 107032 (1.74%) |
