# Supplementary figures and images for: Differences in DNA Methylation Between Disease-Resistant and Disease-Susceptible Chinese Tongue Sole (Cynoglossus semilaevis) Families
Source: Front Genet. 2019 Sep 13;10:847. doi: 10.3389/fgene.2019.00847 (PMC6753864; doi:10.3389/fgene.2019.00847)

Enriched GO Terms  
(NA)

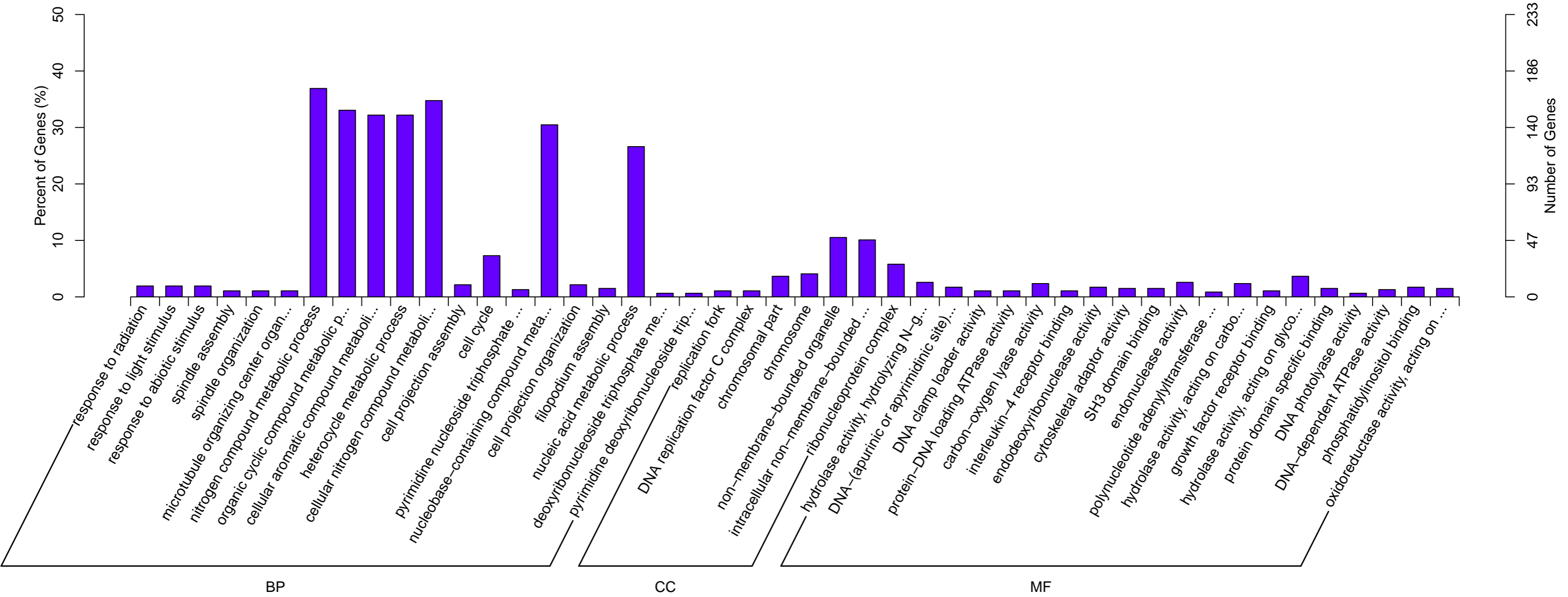

Supplement: Supplementary Figure S1 — DNA methylation levels of mCG, mCHG and mCHH in functional regions of the genome. The blue, green and red features represent the promoter (the 2 kb region upstream of the TSS), exon and intron functional regions, respectively. [file DataSheet_1.zip › Supplementary Fig S7.pdf]

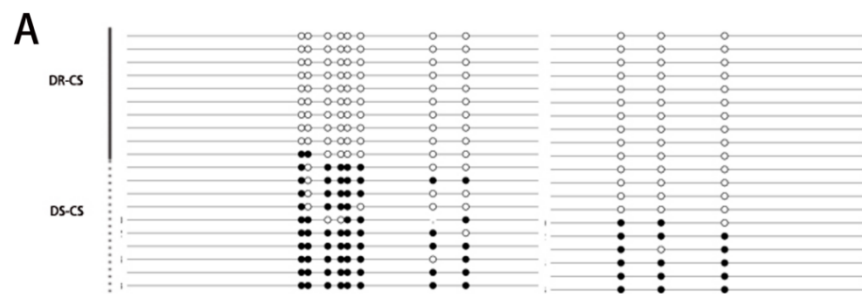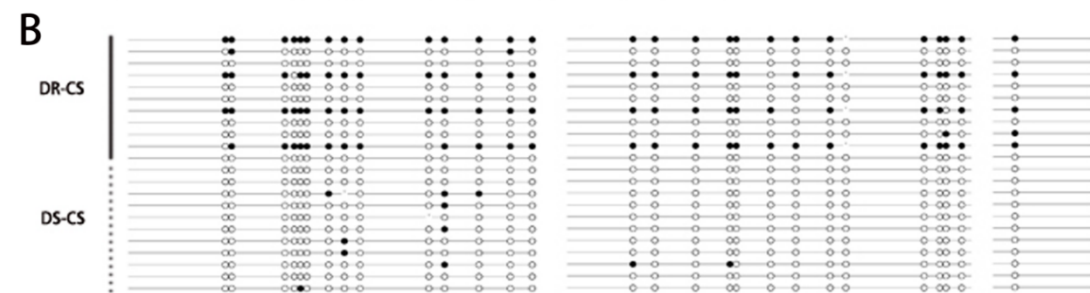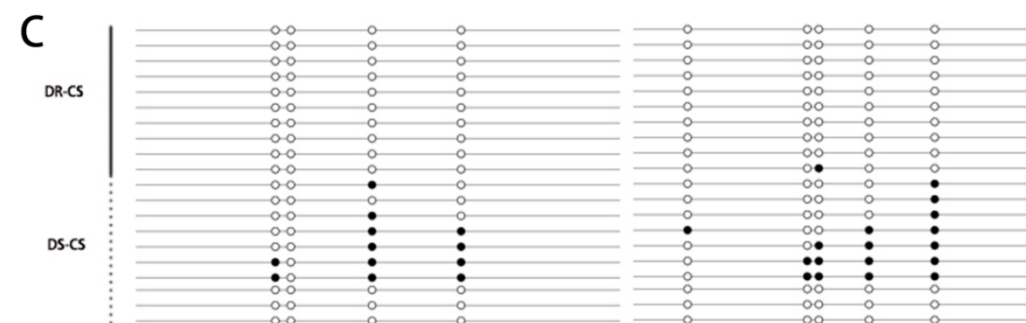

Supplement: Supplementary Figure S1 — DNA methylation levels of mCG, mCHG and mCHH in functional regions of the genome. The blue, green and red features represent the promoter (the 2 kb region upstream of the TSS), exon and intron functional regions, respectively. [file DataSheet_1.zip › Supplementary Fig S8.pdf]

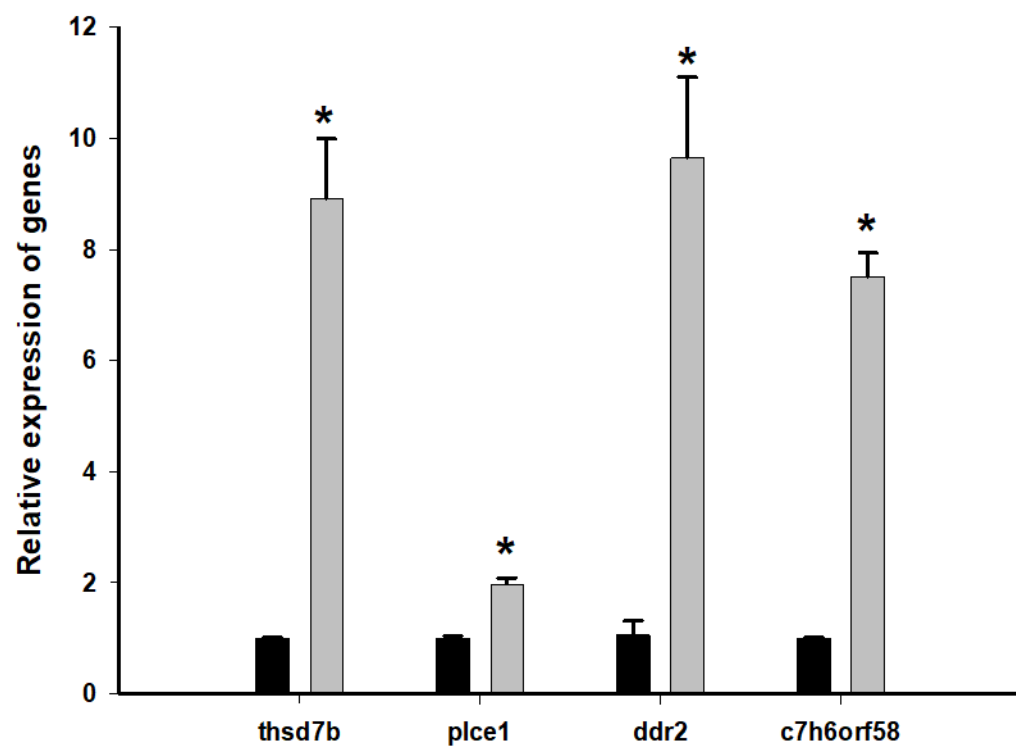

Supplement: Supplementary Figure S1 — DNA methylation levels of mCG, mCHG and mCHH in functional regions of the genome. The blue, green and red features represent the promoter (the 2 kb region upstream of the TSS), exon and intron functional regions, respectively. [file DataSheet_1.zip › Supplementary Fig S9.pdf]

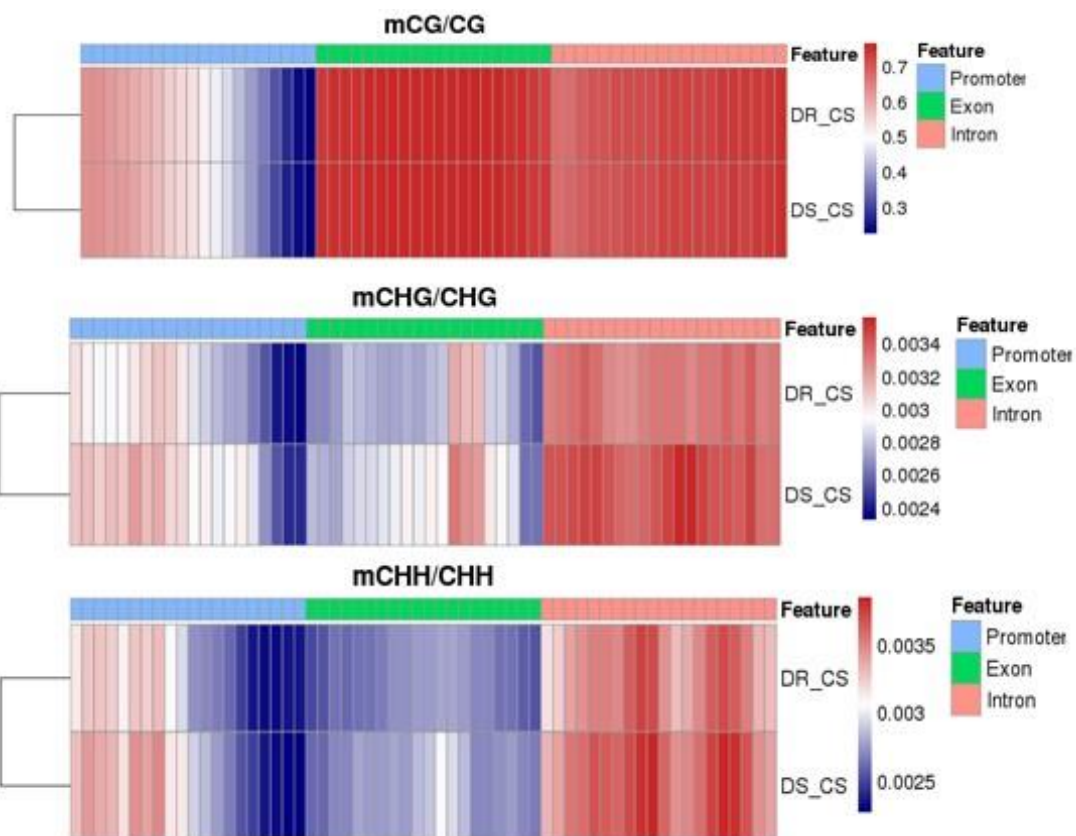

Supplement: Supplementary Figure S1 — DNA methylation levels of mCG, mCHG and mCHH in functional regions of the genome. The blue, green and red features represent the promoter (the 2 kb region upstream of the TSS), exon and intron functional regions, respectively. [file DataSheet_1.zip › Supplementary Fig S1.pdf]

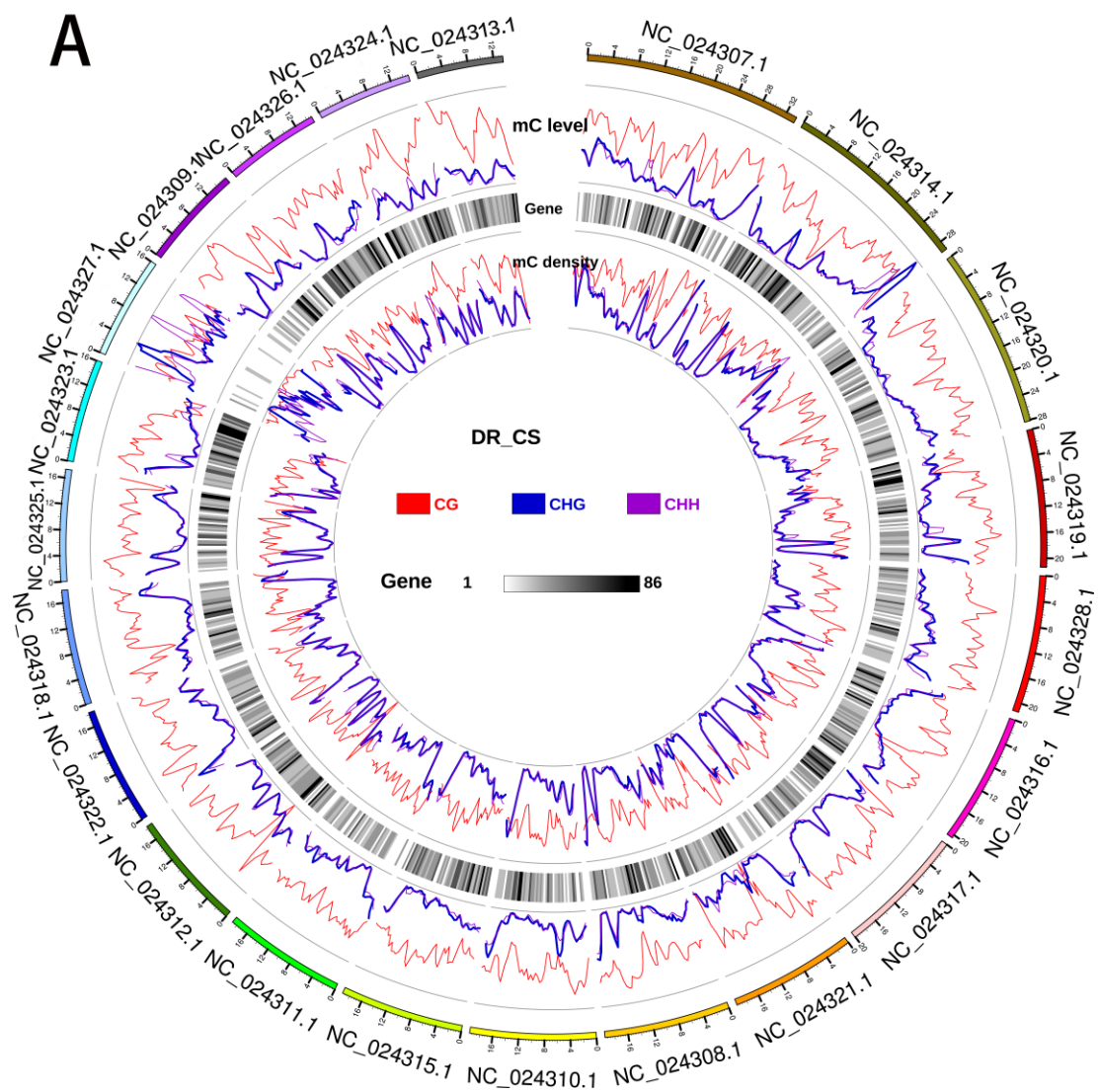

**B**

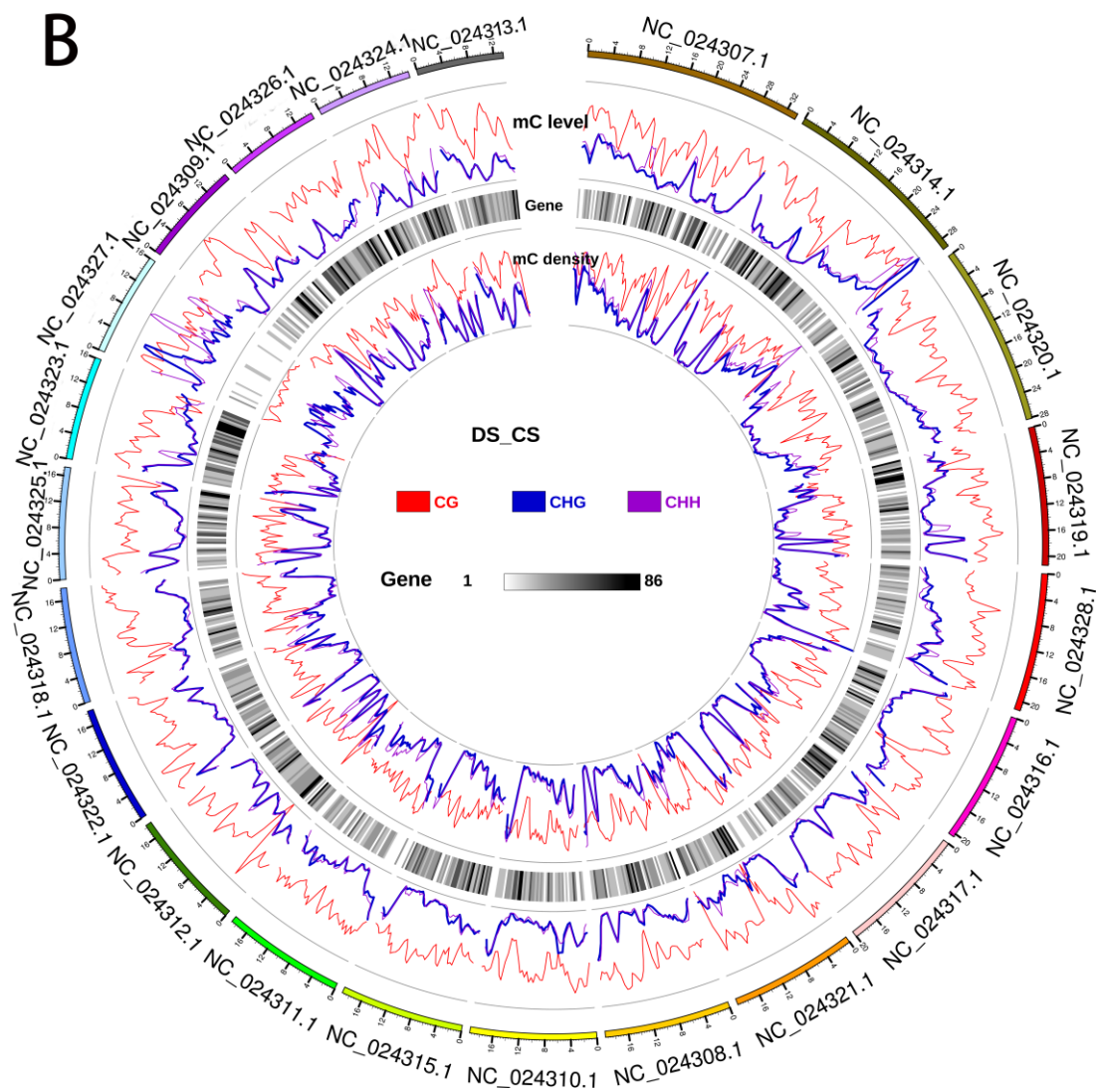

Supplement: Supplementary Figure S1 — DNA methylation levels of mCG, mCHG and mCHH in functional regions of the genome. The blue, green and red features represent the promoter (the 2 kb region upstream of the TSS), exon and intron functional regions, respectively. [file DataSheet_1.zip › Supplementary Fig S2.pdf]

A

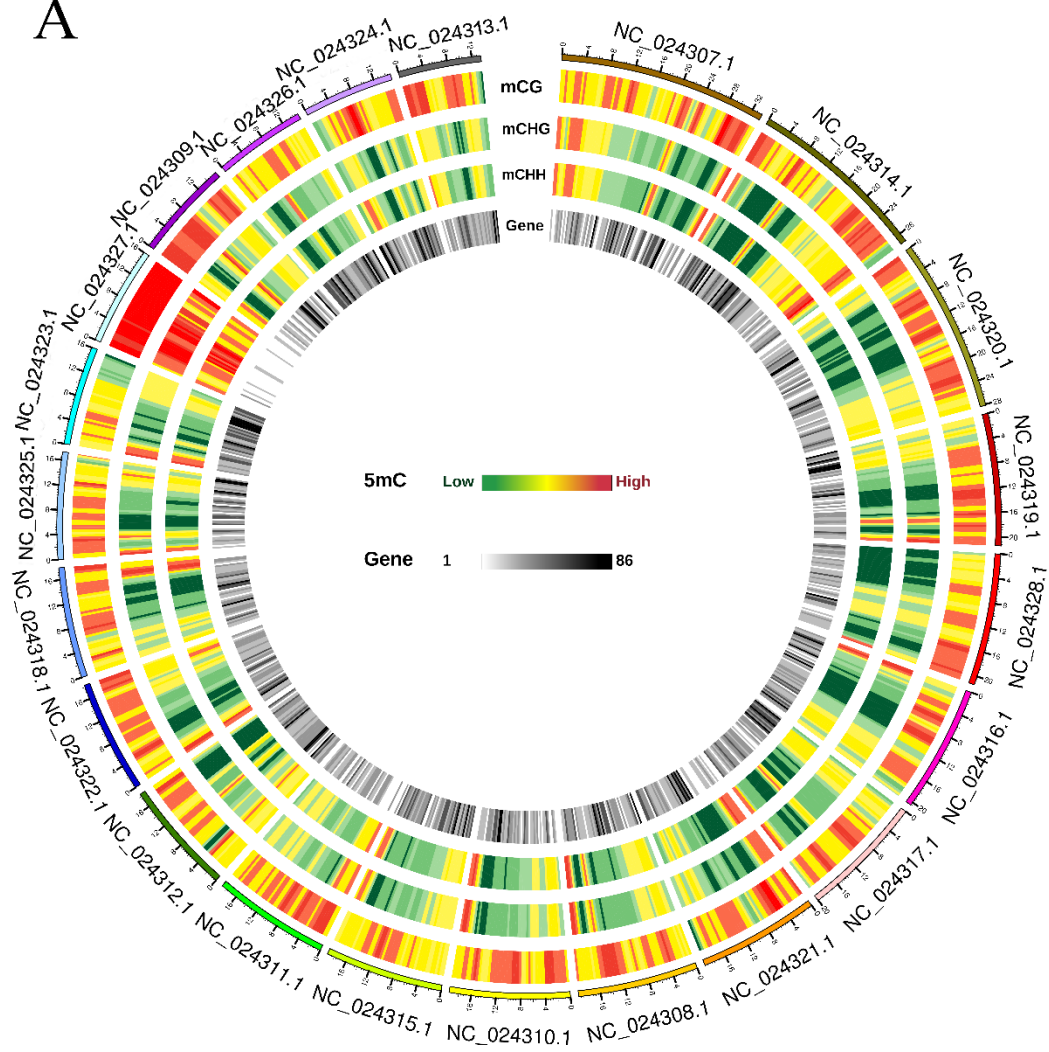

B

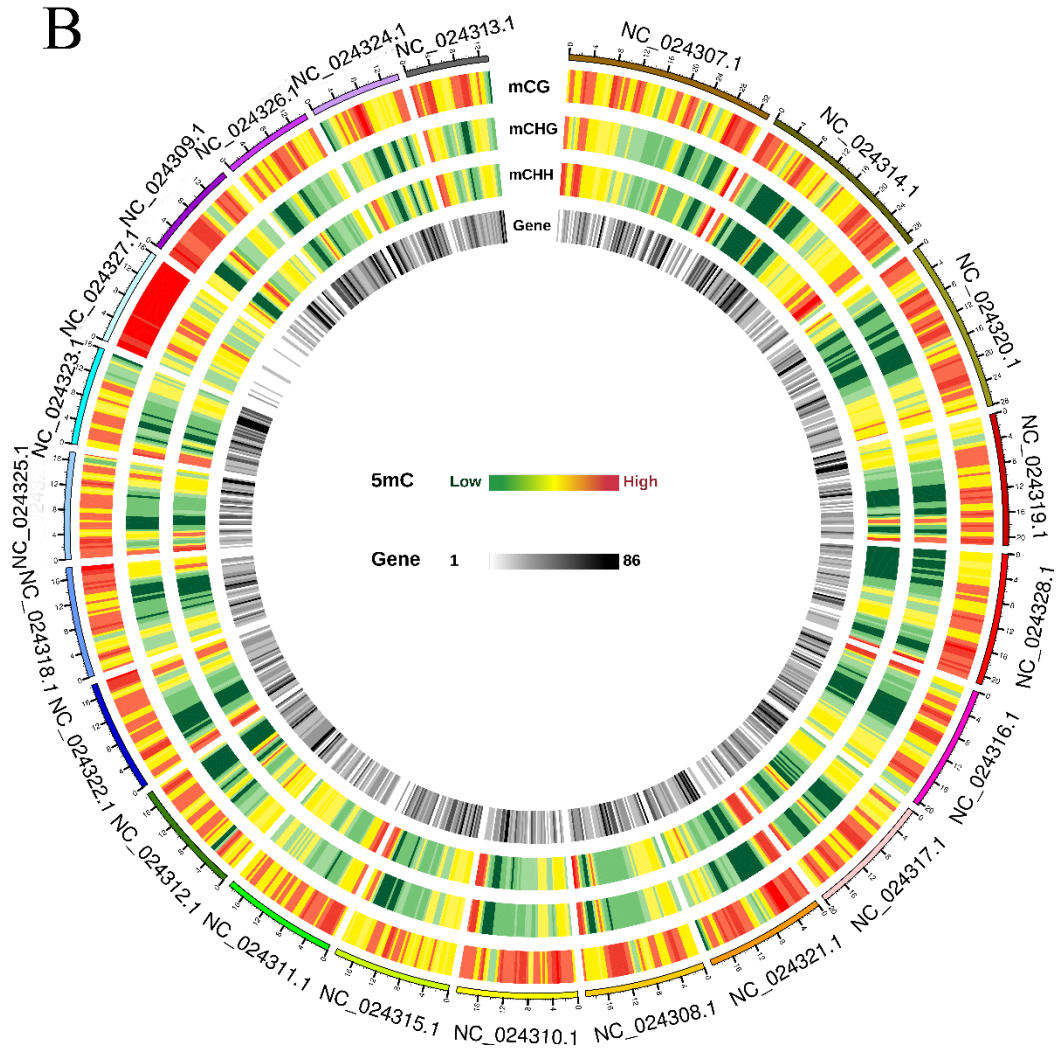

Supplement: Supplementary Figure S1 — DNA methylation levels of mCG, mCHG and mCHH in functional regions of the genome. The blue, green and red features represent the promoter (the 2 kb region upstream of the TSS), exon and intron functional regions, respectively. [file DataSheet_1.zip › Supplementary Fig S3.pdf]

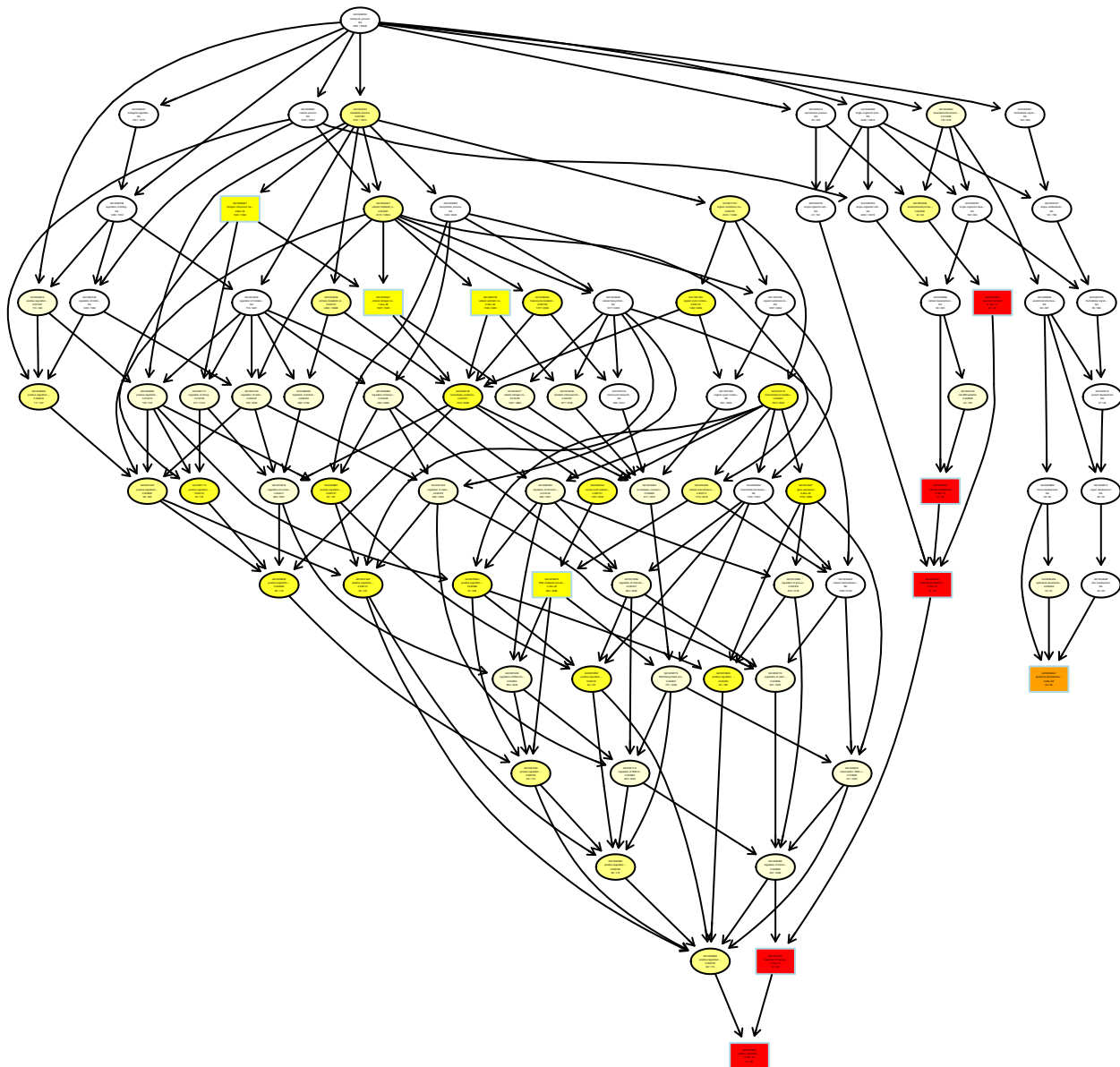

Supplement: Supplementary Figure S1 — DNA methylation levels of mCG, mCHG and mCHH in functional regions of the genome. The blue, green and red features represent the promoter (the 2 kb region upstream of the TSS), exon and intron functional regions, respectively. [file DataSheet_1.zip › Supplementary Fig S4.pdf]

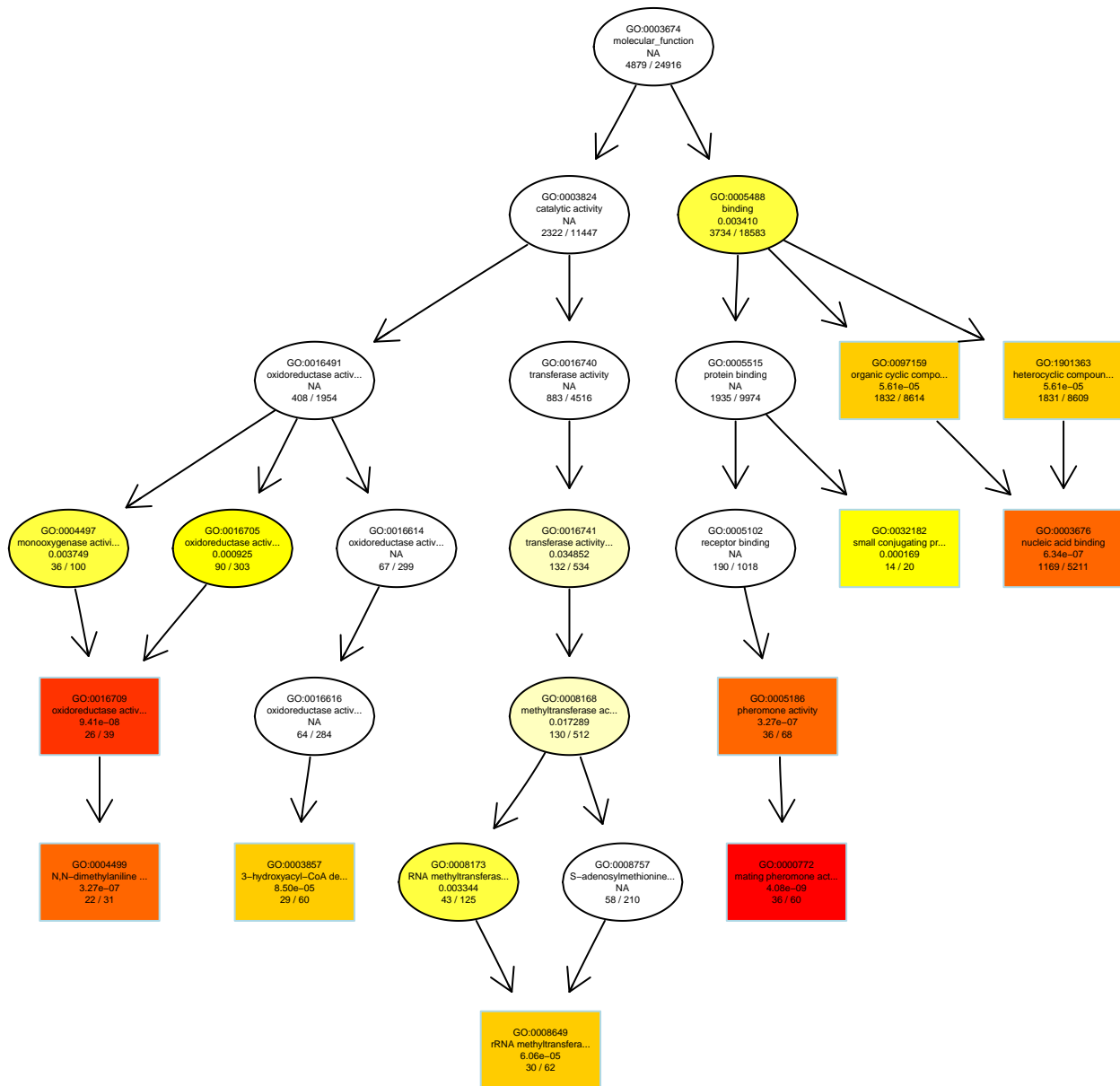

Supplement: Supplementary Figure S1 — DNA methylation levels of mCG, mCHG and mCHH in functional regions of the genome. The blue, green and red features represent the promoter (the 2 kb region upstream of the TSS), exon and intron functional regions, respectively. [file DataSheet_1.zip › Supplementary Fig S5.pdf]

# Enriched GO Terms (NA)

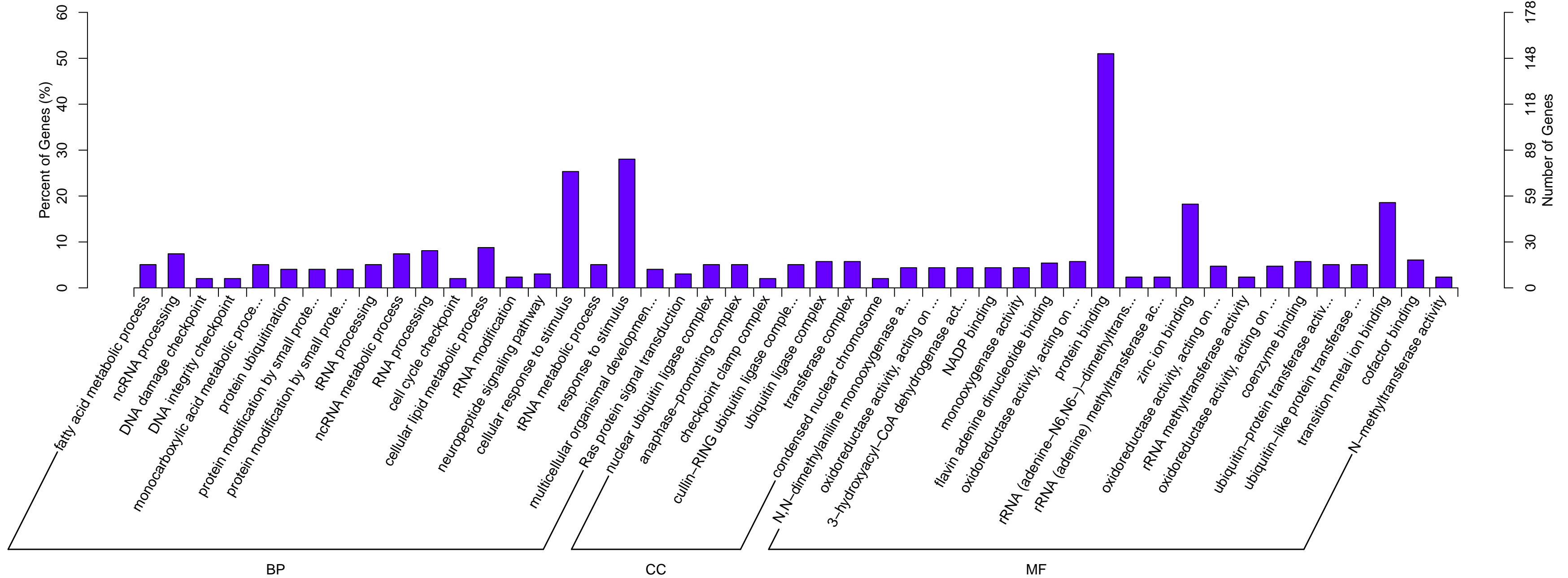

Supplement: Supplementary Figure S1 — DNA methylation levels of mCG, mCHG and mCHH in functional regions of the genome. The blue, green and red features represent the promoter (the 2 kb region upstream of the TSS), exon and intron functional regions, respectively. [file DataSheet_1.zip › Supplementary Fig S6.pdf]
